# Quantitative imaging biomarkers of immune-related adverse events in immune-checkpoint blockade-treated metastatic melanoma patients: a pilot study

**DOI:** 10.1007/s00259-021-05650-3

**Published:** 2021-12-27

**Authors:** Nežka Hribernik, Daniel T Huff, Andrej Studen, Katarina Zevnik, Žan Klaneček, Hamid Emamekhoo, Katja Škalic, Robert Jeraj, Martina Reberšek

**Affiliations:** 1 Department of Medical Oncology, Institute of Oncology Ljubljana, Zaloška 2, SI-1000 Ljubljana, Slovenia; 2 Faculty of Medicine, University of Ljubljana, Ljubljana, Slovenia; 3 Department of Medical Physics, School of Medicine and Public Health, University of Wisconsin-Madison, Madison, WI, USA; 4 University of Wisconsin Carbone Cancer Centre, Madison, WI, USA; 5 Faculty of Mathematics and Physics, University of Ljubljana, Ljubljana, Slovenia; 6 Jožef Stefan Institute, Ljubljana, Slovenia; 7 Department of Nuclear Medicine, Institute of Oncology Ljubljana, Ljubljana, Slovenia; 8 Department of Medicine, School of Medicine and Public Health, University of Wisconsin-Madison, Madison, WI, USA

**Keywords:** Immune-checkpoint inhibitors, Immune-related adverse effects, ^18^F-FDG PET/CT, Quantitative imaging biomarkers

## Abstract

**Purpose:**

To develop quantitative molecular imaging biomarkers of immune-related adverse event (irAE) development in malignant melanoma (MM) patients receiving immune-checkpoint inhibitors (ICI) imaged with ^18^F-FDG PET/CT.

**Methods:**

^18^F-FDG PET/CT images of 58 MM patients treated with anti-PD-1 or anti-CTLA-4 ICI were retrospectively analyzed for indication of irAE. Three target organs, most commonly affected by irAE, were considered: bowel, lung, and thyroid. Patient charts were reviewed to identify which patients experienced irAE, irAE grade, and time to irAE diagnosis. Target organs were segmented using a convolutional neural network (CNN), and novel quantitative imaging biomarkers — SUV percentiles (SUV_X%_) of ^18^F-FDG uptake within the target organs — were correlated with the clinical irAE status. Area under the receiver-operating characteristic curve (AUROC) was used to quantify irAE detection performance. Patients who did not experience irAE were used to establish normal ranges for target organ ^18^F-FDG uptake.

**Results:**

A total of 31% (18/58) patients experienced irAE in the three target organs: bowel (*n*=6), lung (*n*=5), and thyroid (*n*=9). Optimal percentiles for identifying irAE were bowel (SUV_95%_, AUROC=0.79), lung (SUV_95%_, AUROC=0.98), and thyroid (SUV_75%_, AUROC=0.88). Optimal cut-offs for irAE detection were bowel (SUV_95%_>2.7 g/mL), lung (SUV_95%_>1.7 g/mL), and thyroid (SUV_75%_>2.1 g/mL). Normal ranges (95% confidence interval) for the SUV percentiles in patients without irAE were bowel [1.74, 2.86 g/mL], lung [0.73, 1.46 g/mL], and thyroid [0.86, 1.99 g/mL].

**Conclusions:**

Increased ^18^F-FDG uptake within irAE-affected organs provides predictive information about the development of irAE in MM patients receiving ICI and represents a potential quantitative imaging biomarker for irAE. Some irAE can be detected on ^18^F-FDG PET/CT well before clinical symptoms appear.

## Introduction

New cancer treatments with ICI have improved outcomes and altered management strategies for patients with melanoma and a variety of other malignancies. Many trials have shown the safety and efficacy of ICI targeting programmed death-1 (PD-1), programmed death ligand-1 (PD-L1), and cytotoxic T-lymphocyte antigen-4 (CTLA-4) and these agents are now widely implemented treatments for melanoma in both metastatic and adjuvant settings. None of the anti-PDL-1 agents has been approved for treatment of melanoma as single agent or in combination with other ICIs [1–5].

ICI treatments might cause immune-related toxicities, called irAE, where an immune response is generated against healthy tissue. These irAEs can occur in any organ system [6]. Though the exact pathophysiology of irAE is not completely understood, it is believed that they are provoked by immune upregulation and inflammation. Depending on the treatment regimen, irAEs might occur less frequently when compared to cytotoxic chemotherapy-related toxicities [7]. However, some patients might experience higher grades of irAEs that require hospitalization or prolonged treatment and might be life-threatening [8, 9]. Due to the novel mechanism of action, unpredictable nature, and broad usage of ICIs, development of biomarkers capable of early detection and monitoring of irAEs is an area of urgent need [10].

Positron emission tomography/computed tomography with [^18^F]2fluoro-2-deoxy-D-glucose (^18^F-FDG PET/CT) is a sensitive and non-invasive test commonly used in diagnosis, staging, and treatment response evaluation in MM [9, 11, 12]. The combination of ^18^F-FDG PET and CT allows functional and morphological evaluation of the disease and guides clinical decision-making and treatment selection. It is also a very sensitive method for recognizing inflammatory processes that can be reflective of irAE [13–16]. Some preliminary analysis of irAE detection and monitoring via ^18^F-FDG PET/CT has been undertaken [17–19]. None of them perfomed a quantitative assessment of the observed organs.

For melanoma patients receiving ICI, ^18^F-FDG PET/CT is performed for the purpose of disease response assessment. However, incidental findings of increased tracer uptake in off-target organs are sometimes reported. The fact that ^18^F-FDG PET/CT is currently not used to detect irAE may be due in part to the impracticality of performing a manual whole-organ assessment of ^18^F-FDG uptake. Small organs such as the thyroid can be assessed manually with relative ease, but larger organs such as the bowel or lung would require hours of expert physician time to contour manually. We hypothesize that this is a contributing factor to why irAE assessment on ^18^F-FDG PET/CT has thus far been limited to the thyroid [17, 18], or to qualitative assessment only in the bowel [19]. To overcome this hurdle, automated image analysis techniques can be employed. In particular, the segmentation of organs on PET/CT via deep learning-based CNNs has recently become possible [20, 21].

The goal of this study was to identify quantitative imaging biomarkers of irAE development on ^18^F-FDG PET/CT in a cohort of patients with MM who were treated with ICI. MM is specifically selected for this pilot retrospective study as ICI regimens and response evaluation with ^18^F-FDG PET/CT are standard of care treatment for this disease and routinely performed. Other malignancies that utilize ^18^F-FDG PET/CT for response evaluation use ICI combined with other treatment modalities (e.g., cytotoxic chemotherapy or targeted therapy) which makes it difficult to tease out irAE from other treatment-related AEs. We hypothesized that an automated organ segmentation method would be capable of quantifying irAE-susceptible organ inflammation in ^18^F-FDG PET/CT images in metastatic melanoma patients treated with ICI.

## Patients and methods

We conducted a retrospective pilot study, analyzing ^18^F-FDG PET/CT images from patients with metastatic melanoma who were treated per standard of care with ICI (anti-CTLA-4 or anti-PD1) at the Institute of Oncology Ljubljana (OIL), Slovenia (January 2016–January 2019) or at the University of Wisconsin Carbone Cancer Centre (UWCCC), Madison, WI, USA (June 2012–June 2019). None of the patients had an autoimmune disease. All available ^18^F-FDG PET/CT data acquired before and during ICI treatment was collected for review. We determined the date of clinical irAE detection via chart review. If the irAE grade was not explicitly documented in the chart, when possible, irAE grading was assigned retrospectively based on the available clinical course documentation following Common Terminology Criteria for Adverse Events (CTCAE, v.5.0) [22]. Clinical and demographic data were collected from both hospital databases. Clinical and imaging data were anonymized and stored in a secure LabKey database server [23].

The study was approved by the Institutional Review Board Committee of both Institutions (Approval number: 2016–0418 in Madison, USA; ERIDKE-0005/2020 in Ljubljana, Slovenia) and was conducted following the ethical standards defined by the Declaration of Helsinki. At OIL, patients have signed informed consent for treatment and consent allowing the usage of their data for scientific purposes. At UWCCC, the study was approved with a waiver of informed consent.

### PET acquisition

PET scans were primarily performed for immunotherapy treatment response evaluation in melanoma patients. Images were acquired on five PET/CT scanners: GE Discovery 710, GE Discovery STE, GE Discovery IQ, GE Discovery MI (General Electric, Waukesha, WI), and mCT (Siemens, Knoxville, TN). In all cases, the imaging protocol required patients to fast for 6 h prior to injection of the radiotracer and have a blood glucose level below 200 mg/dL (UWCCC) or 6–10 mmol/L (OIL) at the time of the scan. Patients were required to hold all diabetic medication, including metformin, for 6 h prior to radiotracer injection. On the GE Discovery IQ, patients were injected with 259±52 MBq of ^18^F-FDG, while on other scanners, patients were injected with a weight-based dose of 5 (OIL) to 5.2 MBq per kilogram and a minimum 370 MBq (UWCCC) of ^18^F-FDG. Scans were acquired 60±10 min post-injection. For UWCCC patients, the CT used in segmentation was a low-dose CT acquired for attenuation correction. At OIL, CT that meets RECIST analysis needs was acquired according to adjusted protocol including SAFIR reconstruction to minimize dose. Following reconstruction, images were normalized by patient weight and injected dose to compute standardized uptake values (SUV). If available, TOF reconstruction was used.

### ^18^F-FDG PET/CT image analysis

To quantify organ ^18^F-FDG uptake, a CNN was trained to segment the thyroid, lungs, and bowel from the low-dose CT component of patients’ PET/CT imaging data. A CNN was chosen for segmentation for the ability to segment irregular and variable structures and for the ability to successfully segment multiple target structures with very different sizes (e.g., thyroid versus lung) [21]. The network architecture used was DeepMedic, a 3-D, patch-based CNN with multi-resolution pathways [24]. The loss function used was Dice similarity coefficient (DSC). The optimizer was RMSprop [25]. Sixty manual contours of the bowel, lung, and thyroid were produced using a public dataset of *N*=20 patients from the VISCERAL.eu Anatomy3 benchmark [26], and an additional private institutional dataset of *N*=40 patients by an experienced graduate student using 3D Slicer [27]. Labelled data were split 80%/20% (*n*=48/*n*=12) for CNN training/validation. Images were resampled to a cubic 2-mm grid and normalized to have a mean of 0 and variance of 1 within the patient. Data augmentation via histogram shifting, histogram scaling, and random rotation was used to increase the effective training dataset size. The CNN was trained using a workstation with one NVIDIA Titan Xp GPU with 12 GB of memory.

The trained CNN was used to perform inference on the CT component from the ^18^F-FDG PET scans and produce contours of the thyroid, lung, and bowel. The contours were then applied to the PET image to quantify ^18^F-FDG uptake within the three target organs. To determine the ability of PET to detect irAE, percentiles of the distribution of SUV from within each target organ (SUV_X%_) were extracted. Percentiles of the distribution of organ SUV were pursued as potential biomarkers of irAE due to their improved reliability as compared to SUV_max_ [28].

Receiver operating characteristic (ROC) analysis was performed to determine the value of organ SUV percentiles as potential quantitative imaging biomarkers of irAE development. This was done by comparing organ SUV percentiles with the clinical irAE status as determined by chart review. For patients who had multiple ^18^F-FDG PET/CT exams during ICI treatment, the maximum organ SUV percentile value was used as a predictor of irAE. The optimal organ SUV percentile (SUV_OPT%_) was defined to be the percentile that maximized the area under the ROC curve (AUROC) for predicting irAE status (Eq. 1).

(1) $${\text{SUV}}_{\text{OPT}\%}=\underset{x\in {\text{SUV}}_{\text{X}\%}}{\text{argmax}}\text{AUROC}\left(x\right)$$

where SUV_X%_ are the set of percentiles of the distribution of organ SUV. SUV_OPT%_ was measured on all available ^18^F-FDG PET scans and tracked longitudinally to assess if changes in target organ ^18^F-FDG uptake may precede clinical irAE identification.

Target organ ^18^F-FDG uptake was also assessed in patients who did not experience irAE. This was done to establish normal ranges for organ ^18^F-FDG SUV_OPT%_ values against which SUV_OPT%_ values from patients with irAE can be compared. The 95% confidence interval for SUV_OPT%_ was determined for each target organ using the baseline PET images of *N*=15 patients who did not experience any irAE (Eq. 2).

(2) $${\text{CI}}_{95}=\left[\mu -1.96\sigma ,\mu +1.96\sigma \right]$$

where *μ* and *σ* are the mean and standard deviation of baseline SUV_OPT%_ values of patients who did not experience irAE, respectively.

### Statistical analysis

For each target organ (bowel, lung, thyroid), patients were divided into two groups: patients who experienced irAE and patients who did not experience irAE. Differences in SUV metric by irAE status were assessed with Wilcoxon rank-sum tests. *p*<0.05 was considered to be statistically significant. ROC analysis was performed to determine the ability of SUV metrics to detect irAE. Optimal cutoff values for detecting irAE were assigned to maximize the Youden’s index (sensitivity+specificity-1). Image analysis and statistical testing was done using MATLAB R2020b (**The MathWorks, Inc., Natick, MA, USA)**.

## Results

### Patient characteristics

Out of 58 MM patients treated with anti-PD-1 or anti-CTLA-4 ICI, 20 irAE of interest (colitis, pneumonitis, and thyroiditis) were observed on ^18^F-FDG PET/CT scans in 18 (31%) patients: the bowel *N*=6 (11%), lung *N*=5 (9%), and thyroid *N*=9 (16%). Two patients had multiple target organ irAE (one had colitis and pneumonitis, one had thyroiditis and pneumonitis). ^18^F-FDG PET of 15 (26%) patients without irAE were used to establish normal ranges. The remaining 25 (43%) patients had irAE involving organs other than irAE of interest and were considered jointly with the previous group as negative for the purpose of organ-specific irAE identification.

Patient demographics for all 58 patients are summarized in Table 1.

A total of 261 ^18^F-FDG PET/CT exams were collected. The median number of ^18^F-FDG PET/CT exams available per patient was 4 (range: 2, 16). The median time between baseline ^18^F-FDG PET/CT and ICI treatment start was 36 days (range: −119, 102). The median time from ICI treatment start to first follow-up ^18^F-FDG PET/CT was 113 days (range: 2, 245). All US patients had baseline scan. In the Slovenian patient group, one patient did not have a baseline ^18^F-FDG PET/CT (a CT scan was performed instead) and another patient had a baseline ^18^F-FDG PET/CT at a different institution. Neither of these two patients developed irAE of interest.

Median time from ICI treatment start to clinical identification of irAE was 195 days (range: 17, 310) for bowel (*N* =7), 187 days (range: 122, 299) for lung (*N*=3), and 53 days (range: 17, 637) for thyroid (*N*=10). The date of clinical diagnosis of irAE was not available for two irAE seen on PET scan, both irAE occurred in the same patients.

### CNN organ segmentation

The performance of the CNN for segmentation of the three target organs was assessed with DSC and average symmetric surface distance (ASSD) [29]. Validation DSC for the bowel, lung, and thyroid were as follows: 0.87±0.06, 0.98±0.01, and 0.57±0.11, respectively (mean±sd). Corresponding values for ASSD were 2.6±1.2 mm, 0.8±0.3 mm, and 6.8±9.6 mm.

### Quantification of irAE by ^18^F-FDG PET/CT

**Fig. 1:**
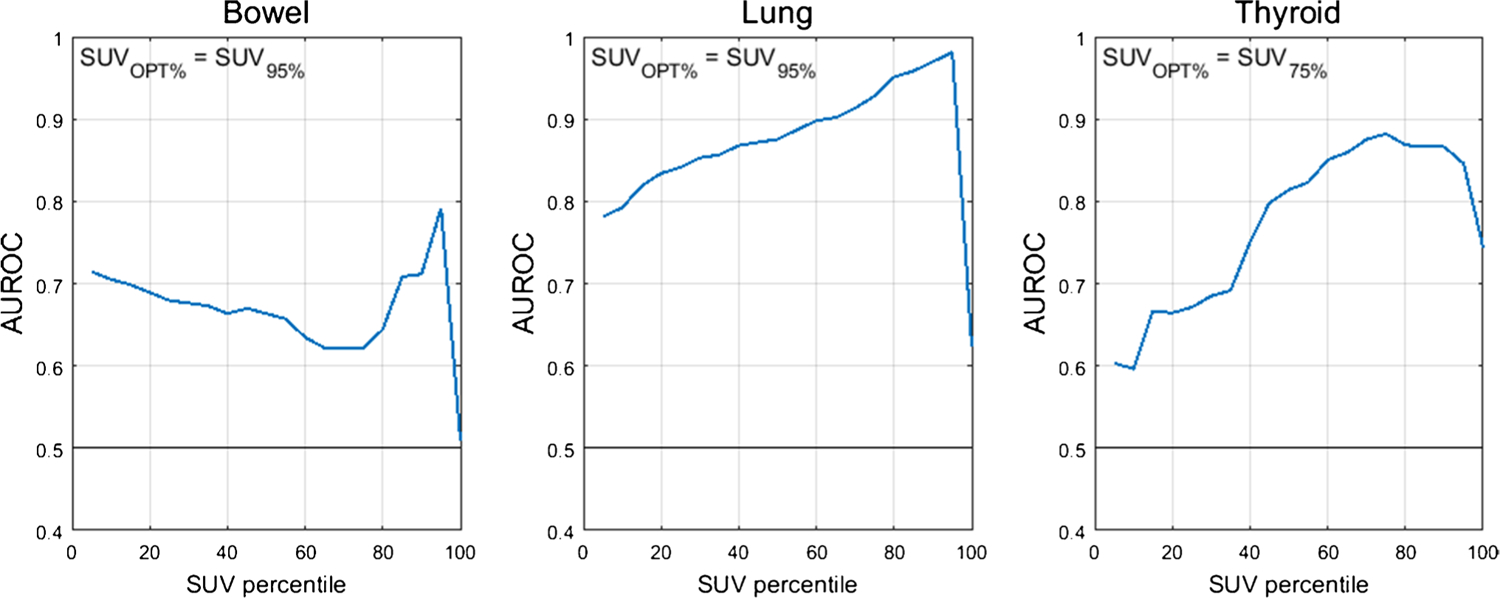
AUROC as a function of organ SUV histogram percentile. The optimal SUV percentile (SUV_OPT%_) is defined as the percentile which maximized the AUROC

**Fig. 2:**
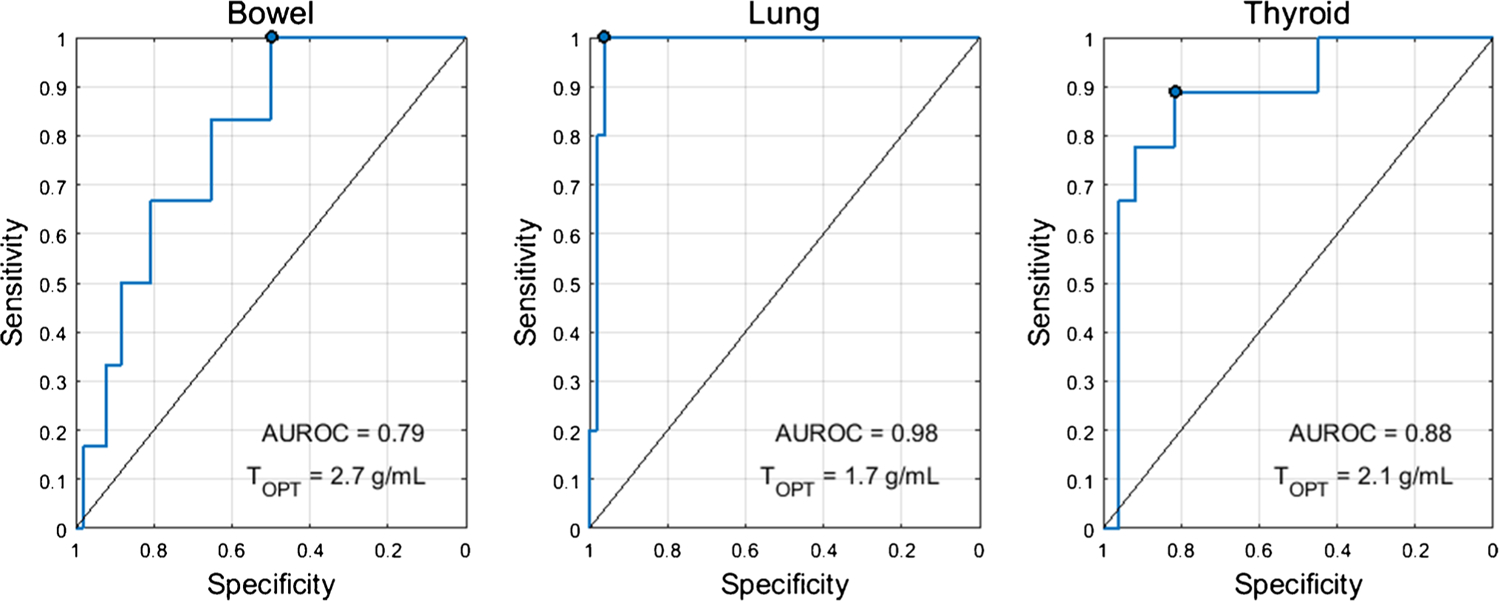
ROC curves for the optimal SUV percentile (SUV_OPT%_) for predicting irAE status in the three target organs. The optimal cutoff (T_OPT_) was defined as the threshold which maximized the Youden’s index (sensitivity+specificity-1). The operating point corresponding to this threshold is marked on the ROC curve

**Fig. 3:**
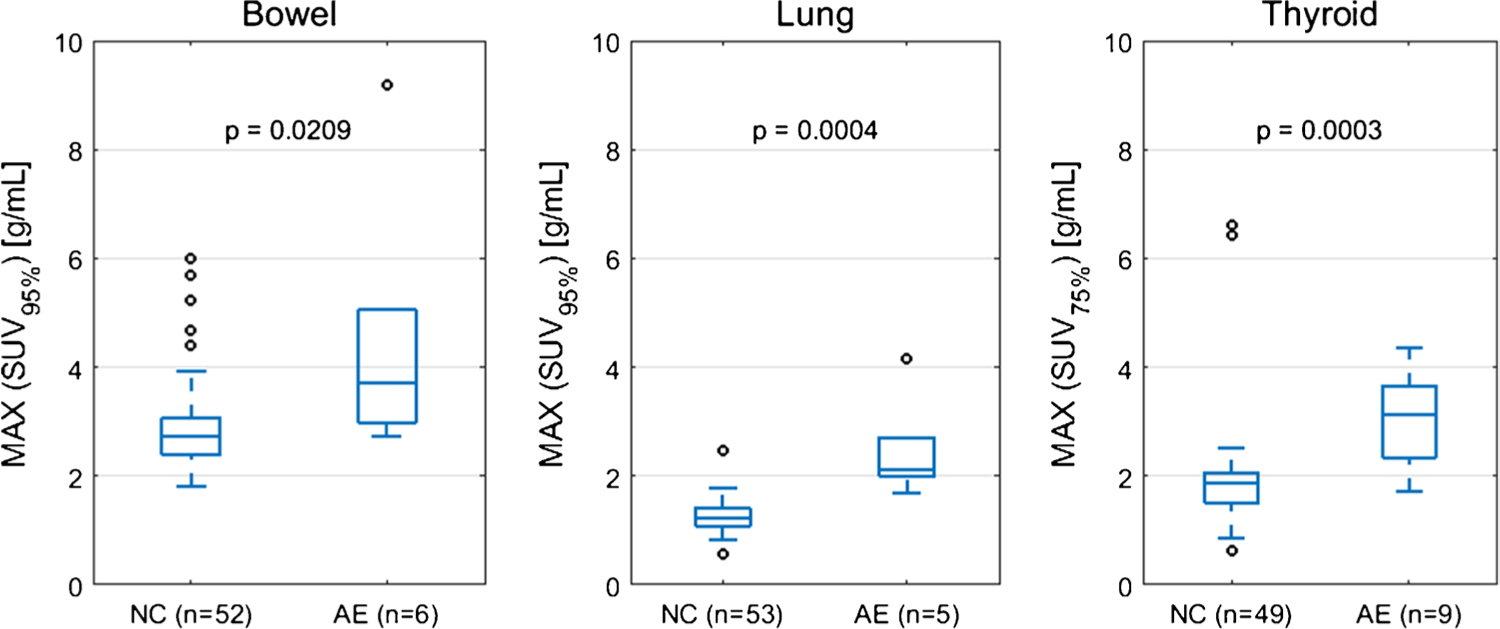
Patient maximum SUV_OPT%_ dichotomized by irAE status (normal control NC versus adverse event AE). The maximum SUV_OPT%_ value is taken for each patient from the set of all on-treatment PET scans. *p*-value from Wilcoxon rank-sum test

The optimal SUV percentiles (SUV_OPT%_) for the three target organs were as follows: bowel=SUV_95%_, lung=SUV_95%_, thyroid=SUV_75%_. AUROC as a function of SUV percentile is shown in Fig. 1. The AUROC for SUV_OPT%_ to predict irAE status was 0.79 for bowel, 0.98 for lung, and 0.88 for thyroid. The optimal cutoff for SUV_OPT%_ was 2.7 g/mL for bowel, 1.7 g/mL for lung, and 2.1 g/mL for thyroid. Sensitivity and specificity at the optimal cutoff were 1.00 and 0.49 for bowel, 1.00 and 0.96 for lung, and 0.89 and 0.81 for thyroid. The ROC curves for SUV_OPT%_ are shown in Fig. 2. SUV_OPT%_ of all target organs was significantly higher for patients with irAE in that organ than for patients without irAE (Wilcoxon rank-sum test, *p*<0.05) (Fig. 3).

### irAE detection by ^18^F-FDG PET/CT versus clinical detection

**Fig. 4:**
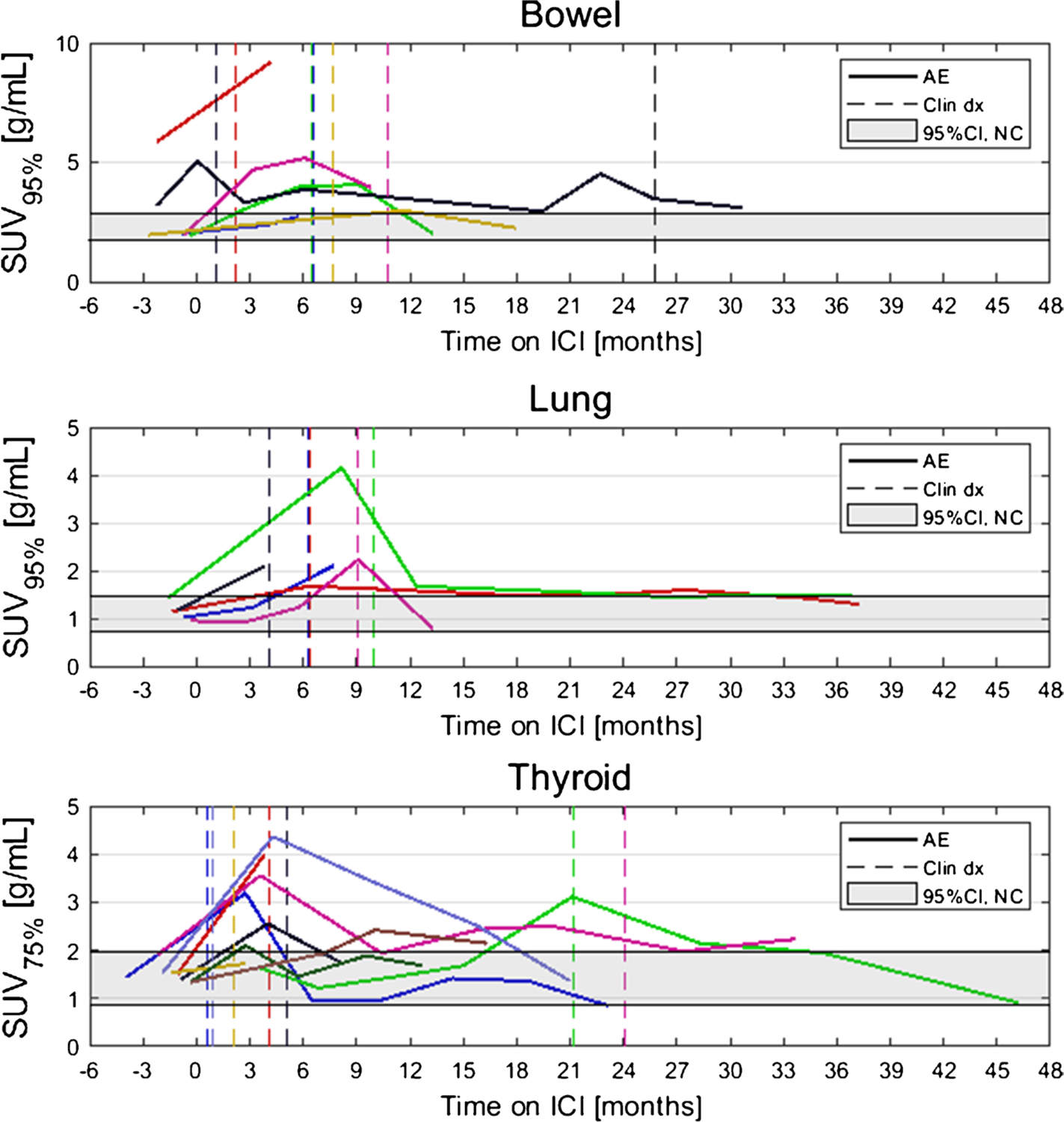
Longitudinal SUV_OPT%_ for patients who experienced irAE in bowel, lung, and thyroid. Dashed vertical lines indicate dates of clinical irAE identification, when available. The grey band indicates the 95% confidence interval for organ SUV_OPT%_ of patients who did not experience irAE

**Fig. 5:**
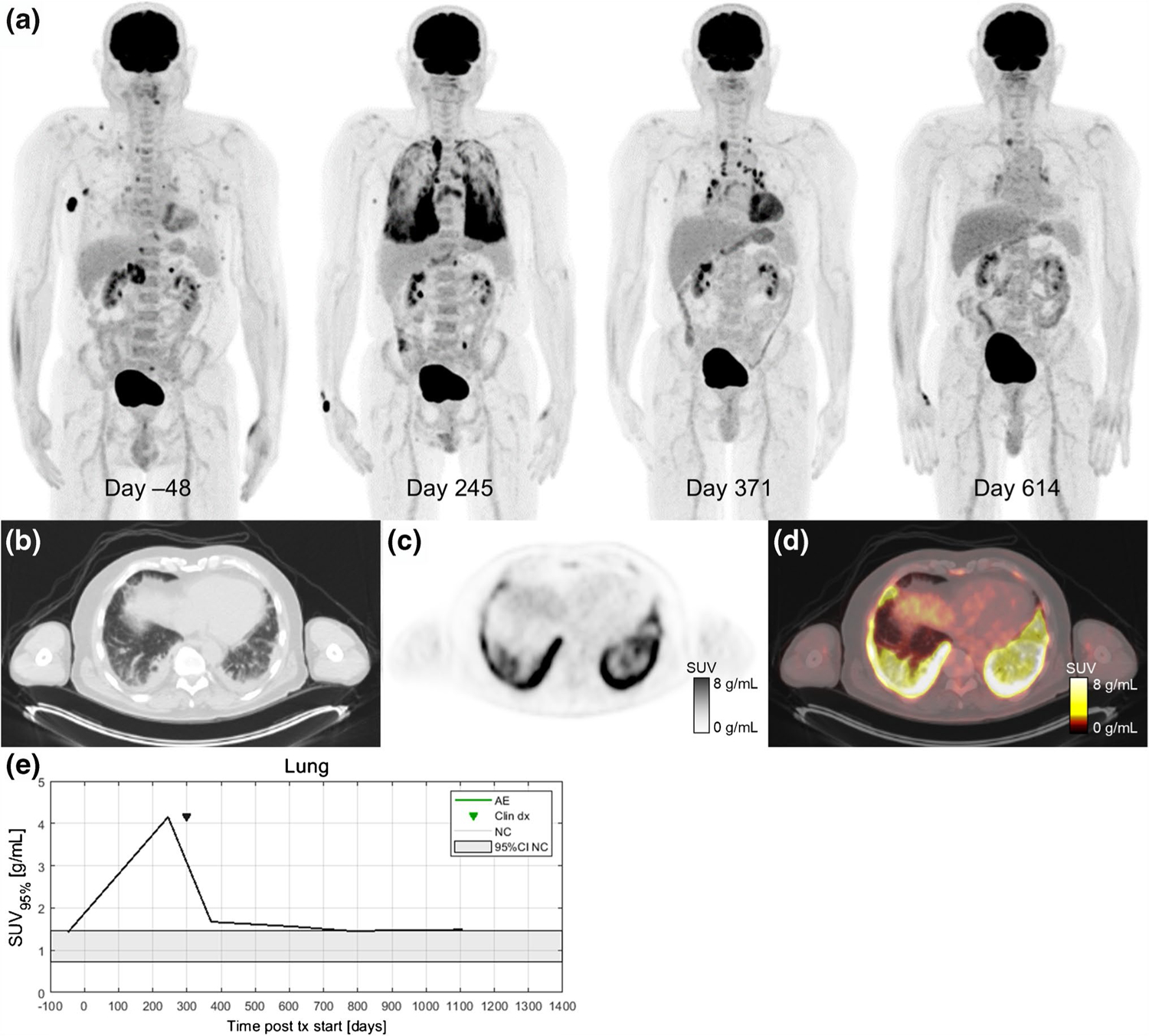
A 67-year-old patient with metastatic BRAF V600E-mutated melanoma with metastases to soft tissue, adrenal gland, and bone receiving pembrolizumab treatment in the first-line setting. Serial ^18^F-FDG PET maximum intensity projections were obtained before and during ICI therapy (**a**). After 8 months of immunotherapy (day 245), a follow-up ^18^F-FDG PET/CT scan showed a partial metabolic response in all metastatic lesions and diffusely elevated ^18^F-FDG uptake in multiple lung lobes. Axial slices at the lung base of the day 245 imaging study on CT (**b**), PET (**c**), and fused PET/CT (**d**). The patient reported only mild dyspnea and no other symptoms at his next oncology follow-up visit. Additional workup included a high-resolution CT scan and bronchoscopy. Immune-related pneumonitis was histologically proven on day 275, and the patient started treatment with systemic glucocorticoids (0.5 mg/kg/day methylprednisolone p.o.). Pneumonitis improved significantly after 3 months of corticosteroid treatment (day 371). ^18^F-FDG PET/CT follow-up performed 8 months later (day 614) shows complete response and normal ^18^F-FDG uptake within the lungs and lymph nodes. In this case, increased ^18^F-FDG uptake in the lungs was detected before the clinical presentation of irPneumonitis and ^18^F-FDG uptake gradually decreased as the symptoms improved on systemic corticosteroid therapy (**e**)

**Fig. 6:**
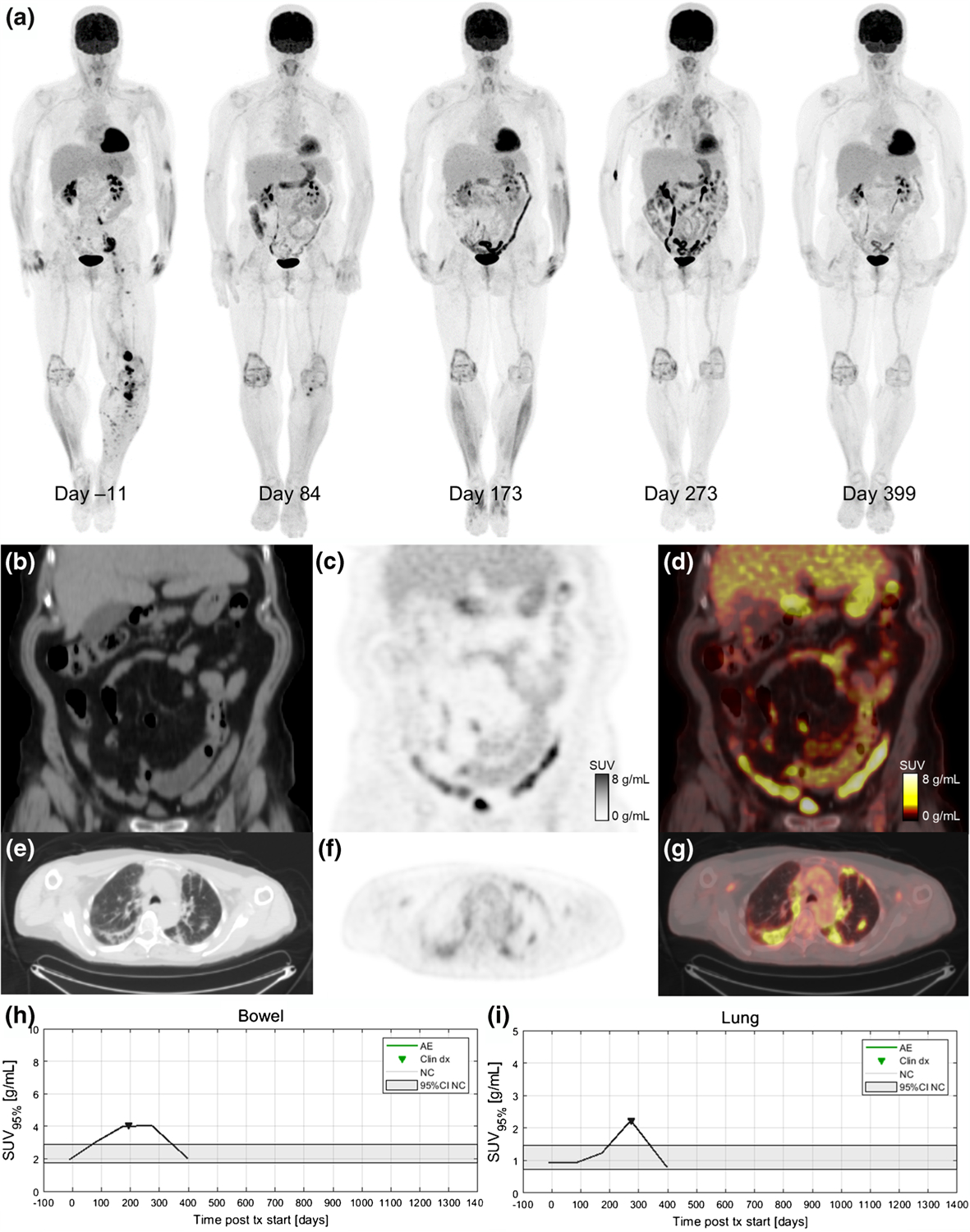
A 68-year-old patient with metastatic BRAF-wild-type melanoma who was treated with ipilimumab/nivolumab combination. Serial ^18^F-FDG PET maximum intensity projections during ICI treatment are shown in (**a**). The baseline image (day 11) shows sites of metastatic disease in the pelvis and left lower extremity. Day 84 after treatment initiation, the patient has a near complete response (CR) of their disease and a moderate increase in ^18^F-FDG uptake in the bowel (SUV_95%_=3.1 g/mL). Day 173 scan indicates continued disease response but marked increased bowel uptake (SUV_95%_=4.0 g/mL) is apparent. Coronal slices from the day 173 study are shown for CT (**b**), PET (**c**), and fused PET/CT (**d**). The patient developed a grade 3 rash and was started on systemic corticosteroids 3 weeks before the day 173 scan. The patient was hospitalized on day 195 due to diarrhea with blood in the stool (grade 3 colitis) and irColitis was confirmed via colonoscopy and biopsy. Day 273 scan shows continued response and elevated bowel uptake as well as diffuse increase tracer uptake in the lungs indicating irPneumonitis. Axial slices from the day 273 imaging study are shown for CT (**e**), PET (**f**), and fused PET/CT (**g**). Day 399 scan shows ongoing disease response, partial resolution of irColitis, and complete resolution of irPneumonitis. The patient had a complete clinical resolution of irColitis after completing a course of corticosteroid therapy with a slow taper in addition to a course of Budesonide treatment. Quantification of organ ^18^F-FDG uptake demonstrated that elevated uptake in the bowel preceded clinical diagnosis of irColitis (**h**) and elevated uptake in the lung corresponded with the time of clinical diagnosis of irPneumonitis (**i**)

**Fig. 7:**
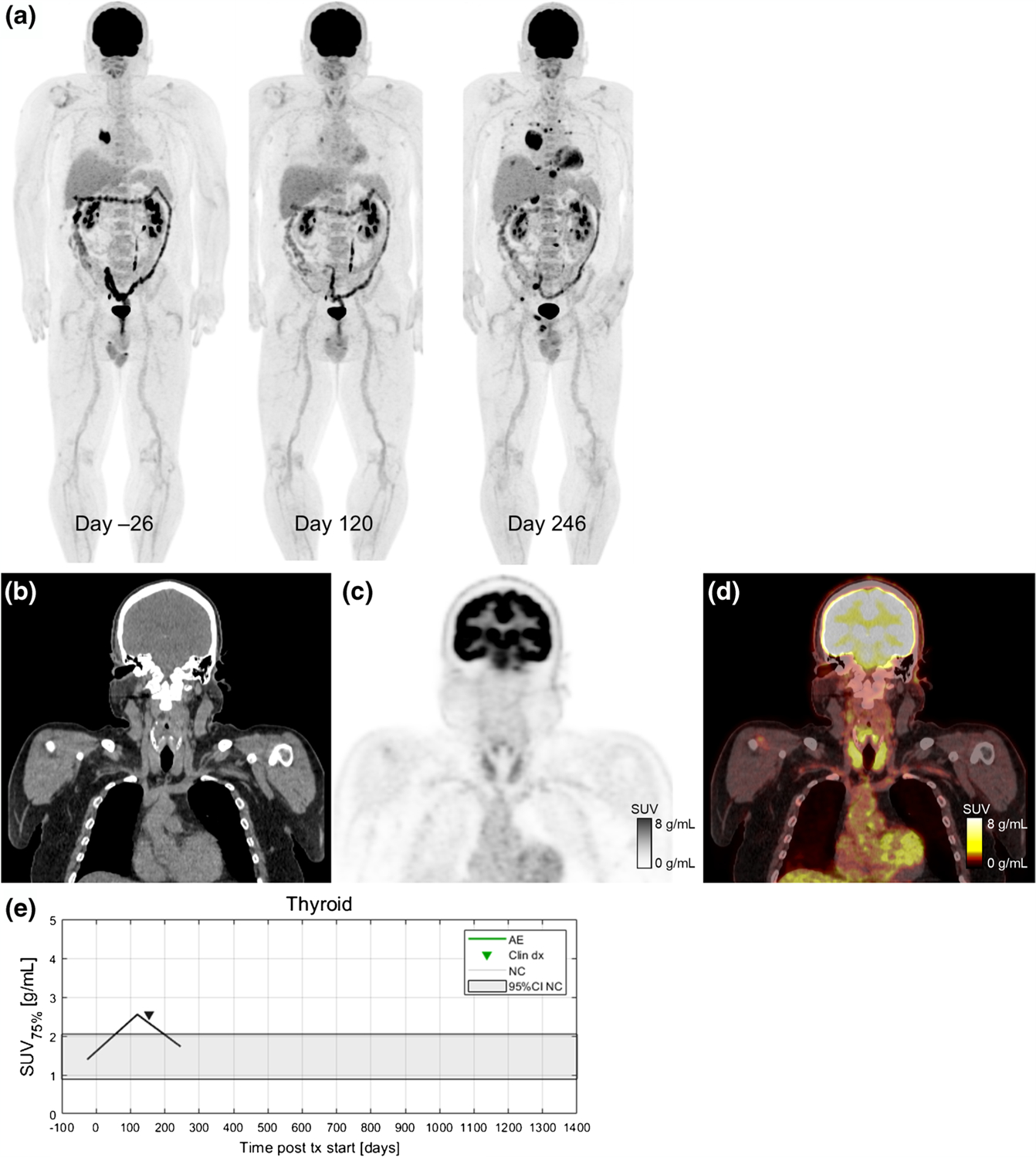
A 54-year-old patient with metastatic BRAF wild-type melanoma who developed immune-related thyroiditis during treatment with immunotherapy with pembrolizumab. Serial ^18^F-FDG PET maximum intensity projections before starting and during ICI therapy are shown in (**a**). The baseline scan from the day 26 shows metastases in soft tissues and lung (both histologically proven). First follow-up scan (day 120) is indicating partial metabolic response in all metastatic lesions and increase in ^18^F-FDG uptake in the thyroid gland (SUV_75%_=2.5 g/mL). On day 154, patient was diagnosed with irThyroiditis according to thyroid hormone laboratory results and endocrinologist’s examination. Next ^18^F-FDG PET/CT (day 246) shows a marked progression disease with new metastatic lesions in bones, liver, lung, right adrenal gland, and soft tissues with decreased ^18^F-FDG uptake in the thyroid gland (SUV_75%_=1.7 g/mL). Coronal slices from day 120 are shown for CT (**b**), PET (**c**), and fused PET/CT (**d**). Increase in ^18^F-FDG uptake in the thyroid gland was seen on PET 34 days before clinical detection (**e**)

Dates of clinical irAE detection were collected via medical records review. Longitudinal time series of SUV_OPT%_ for patients with irAE are shown in Fig. 4 with dates of clinical irAE detection indicated with dashed vertical lines, when available. Multiple cases of target organ ^18^F-FDG uptake increasing outside of normal ranges prior to clinical diagnosis were observed, but due to the retrospective nature of the data collection, no formal statistical comparison of irAE detection timing was made. Images of three patients who experienced irAE of the lung, combination irAE of the lung and bowel, and thyroid are highlighted in Fig. 5, Fig. 6 and 7, respectively.

### Normal ^18^F-FDG uptake in irAE-affected organs

To determine normal ranges for target organ SUV_OPT%_ values, a subcohort of *N*=15 patients who did not experience any irAE was identified. Baseline ^18^F-FDG PET/CT of these patients were analyzed, and SUV_OPT%_ values for each target organ were calculated. From these, a confidence interval for normal SUV_OPT%_ was constructed as defined by Eq. 2. The 95% confidence intervals for SUV_OPT%_ for patients without irAE in the three target organs were as follows: bowel CI_95,SUV95%_=[1.74 g/mL, 2.86 g/mL], lung CI_95,SUV95%_=[0.73 g/mL, 1.46 g/mL], and thyroid CI_95,SUV75%_=[0.86 g/mL, 1.99 g/mL]. Confidence intervals for normal SUV_OPT%_ are shown as horizontal grey bands in Fig. 4.

## Discussion

This is the first study to propose a quantitative imaging analysis of irAE development based on ^18^F-FDG PET/CT imaging. We hypothesized that patients with melanoma who were treated with ICIs and experience irAE would demonstrate increased ^18^F-FDG uptake in the involved organ at the time of clinical irAE diagnosis. In our cohort, patients who experienced immune-related thyroiditis, pneumonitis, or colitis demonstrated increased ^18^F-FDG uptake in the involved organs. This increased uptake could be quantified and monitored longitudinally utilizing an automated image analysis platform that employs CNN-based whole-organ segmentation. Furthermore, elevated ^18^F-FDG uptake preceded clinical detection of irAE in several cases, indicating that ^18^F-FDG PET/CT might provide valuable information in the early detection and management of irAE. This represents an automatic quantitative procedure that is reproducible, non-subjective, and provides information not available by visual analysis only. It provides additional quantitative information for both, radiologist/nuclear medicine to facilitate faster and more accurate informative reads, as well as to a treating physician about the extent of inflammation (e.g., pattern of inflammation, intensity of inflammation). As currently no clinical biomarkers for the development of irAE exists, clinicians must rely on laboratory tests and often perform invasive procedures such as biopsies; quantitative imaging biomarkers would therefore provide welcome additional tool for improved patient management.

We selected three ICI patients who were imaged regularly by ^18^F-FDG PET/CT to highlight as case studies of irAE detection by PET (Fig. 5, 6 and 7). Delay in irAE diagnosis might cause more severe symptoms, which are harder to reverse and could turn into life-threatening situations. Increased radiotracer uptake in the scans preceded the symptomatic clinical diagnosis. In the case of the patient highlighted in Fig. 5, the patient had increased ^18^F-FDG uptake in the lungs on day 245 scan (SUV_95%_=4.1 g/mL) while having minimal symptoms of pneumonitis. This radiographic finding allowed the patient to have all necessary diagnostic procedures performed to confirm irAE, including bronchoscopy. For the patient highlighted in Fig. 6, increasing bowel ^18^F-FDG uptake is seen on the day 84 scan (SUV_95%_=3.1 g/mL) and day 173 scan (SUV_95%_=4.0 g/mL). Immune-related colitis was not clinically diagnosed until 3 weeks later when the patient was hospitalized and underwent colonoscopy with biopsy. This patient also experienced immune-related pneumonitis on day 273, which can be seen as bilateral, diffuse, elevated ^18^F-FDG uptake in the lungs (SUV_95%_=2.2 g/mL). Both patients had a complete resolution of their irAE after receiving systemic corticosteroids, while they continued to have a favorable treatment response in their melanoma.

For identifying irAE, SUV_95%_ was the optimal SUV percentile for the bowel and lung; however, for the thyroid, SUV_75%_ performed best. This difference in the optimal SUV percentile is likely due to the difference in inflammation patterns in different organs. For example, inflammation in thyroid is often relatively uniform throughout the organ volume, and so the SUV histogram is shifted up uniformly in patients with thyroid AE. This is further supported by the relatively small difference in AUROC for thyroid AE classification in the range of SUV_65%_ to SUV_95%_ seen in Fig. 1. In contrast, irAE findings in the lung and bowel are often localized to a small fraction of the organ volume (e.g., a single segment of bowel), so elevated uptake is only reflected in the top few percent of the organ volume. Additionally, the lower segmentation performance of thyroid, being a smaller organ, may also be a contributing factor for reducing the optimal SUV percentile, as mis-segmentation can impact SUV percentile quantification [28]. To further justify our methodology, the metric we used for irAE identification — percentiles of the distribution of organ SUV — is less sensitive to segmentation error than other non-histogram SUV metrics, where only a few mislabelled out of distribution pixels can significantly change the final value. The diversity of scanner and treatment setting in our multicenter study improves the robustness of this analysis by including factors that could potentially impact the SUV percentile calculations.

There are several previous case reports of radiographic findings associated with irAE on ^18^F-FDG PET/CT [30]. In the case of thyroiditis, intense diffuse radiotracer uptake in the thyroid was reported in previous studies [31]. In a small retrospective study of lung cancer patients, increased radiotracer uptake in ^18^F-FDG PET was found to predict thyroiditis even before laboratory findings indicating changes in the thyroid function [17]. Both of these studies relied on manual segmentation of the thyroid for quantification. In our pilot study, we also observed elevated thyroid ^18^F-FDG uptake as quantified by SUV_75%_ in patients who experienced thyroiditis. For colitis, ^18^F-FDG PET was prospectively studied in a cohort of 100 metastatic melanoma patients for colitis/diarrhea [19]. In this study, the determination of PET-colitis was made based on the presence of diffuse, clearly elevated tracer uptake in the colon as interpreted by a radiologist. However, no quantification of colonic ^18^F-FDG uptake was performed. They found a significant correlation between PET-colitis and clinical presentation of diarrhea. In contrast to these studies, our method for irAE detection does not require manual organ segmentation, nor does it rely on radiologist interpretation. Additionally, our study uses optimized metric, percentiles of the SUV distribution, to detect irAE. Our study is also the first to perform a quantitative analysis of ^18^F-FDG uptake of the lung and bowel and evaluate its association with the timing of clinical presentation of irAE in those organs.

For our proposed imaging biomarkers of irAE, the sensitivity and specificity reported were for the cutoff which maximized the sum of sensitivity and specificity. However, depending on clinical need, a different operating point may be more appropriate. The high sensitivity may be traded for increased specificity. This is especially salient in the case of the bowel, where the reported operating point has 100% sensitivity, but only 49% specificity. Instead, operating points of 83% sensitivity/64% specificity or 66% sensitivity/81% specificity could be used for bowel as seen on the ROC curve in Fig. 2.

Another important possible confounding factor in the bowel is the antidiabetic medication metformin that can cause diffuse radiotracer uptake in the bowel. Withholding metformin for 6 h prior to the exam in order to avoid hypoglycemia is not sufficient to reduce the influence of metformin on radiopharmaceutical biodistribution in the bowel. In our cohort, 5/58 (9%) patients had metformin listed in their medication list in the electronic health record (EHR) at the time of ^18^F-FDG PET/CT imaging. None of the patients receiving metformin experienced irColitis; however, 3/5 (60%) demonstrated elevated bowel uptake above the established normal range on at least one ^18^F-FDG PET/CT scan. This is one factor which contributed to decreasing the specificity of SUV_95%_ as a biomarker of irColitis. Additionally, high variation in physiological ^18^F-FDG uptake not related to metformin usage may also contribute to the reduced specificity.

Quantification of ^18^F-FDG uptake in relevant organs for patients undergoing ICI could improve personalized management of these patients. The use of imaging biomarkers could help with proactively managing the treatment-related AE and direct when to hold or discontinue treatment before severe clinically noticeable symptoms appear. The use of accurate quantification could help to detect subclinical inflammation and guide the clinicians to be more vigilant in cases with evidence of high ^18^F-FDG uptake scores. There is a possibility of a subclinical increased inflammation in the target organs which might increase the ^18^F-FDG uptake in patients who did not experience clinically diagnosed irAE. This would have increased the mean baseline tracer uptake. However, this subclinical increase in tracer uptake does not impact the treatment course and was therefore not considered irAE.

Many unanswered questions need to be addressed in future studies. The optimal timing of ^18^F-FDG PET during ICI treatment for early detection of irAEs is currently unknown. The timing of irAE development varies widely, and an irAE can even occur after discontinuation or completion of ICI therapy [32]. Perhaps a quantitative analysis of an early timepoint ^18^F-FDG PET/CT (e.g., at 1 month after ICI treatment initiation) could identify patients who are at greater risk for irAE, as ^18^F-FDG PET/CT after 1 month has been shown to be predictive of patient response to ICI [11]. Furthermore, quantitative analysis of irAE severity and the extent of organ involvement could help to decide if ICI rechallenge could be considered with a reasonably low risk of irAE recurrence. A recent retrospective study reported a 29% recurrence rate of the same irAE after ICI rechallenge [33]. Possibly, ^18^F-FDG PET/CT quantification could help us predict which patients are at higher risk for recurrence of irAE.

In this study, we quantified irAE of the lung, bowel, and thyroid. However, it is known that irAE can affect practically any organ, some of them being very rare but often life threatening [6, 8, 9]. Due to their rarity, a large study cohort would be required to identify imaging biomarkers of irAE in these organs. On the other hand, some irAE are common and can be readily diagnosed clinically, for example, cutaneous irAE (rash, pruritis), which are readily apparent without imaging.

There are several limitations of this study which should be discussed. First, this study was conducted in a retrospective cohort of patients. Due to retrospective nature of the study, some data on irAE was collected by reviewing the available medical documentation and was limited for some patients. Also, there was a variability of timing of ^18^F-FDG PET/CT assessments based on the clinical course of the disease, clinicians’ decision, and mainly for disease status and response assessment and not focused on the irAE assessment. Prospective trials with larger groups of patients, more regimented ^18^F-FDG PET/CT imaging and data collection, and prospective irAE grading and tumor response assessment are needed in the future [34]. Despite these limitations, we provided preliminary data on the process of development of an AI-supported platform capable of quantitative assessment of irAE using ^18^F-FDG PET/CT scans.

## Conclusions

Molecular imaging with ^18^F-FDG PET/CT is a useful tool for detecting and monitoring irAE in melanoma patients receiving ICI. Elevated ^18^F-FDG uptake in the lung, bowel, and thyroid is indicative of the development of irAE and may precede clinical detection of irAE. Early detection of irAE makes it possible to manage irAEs proactively and before development of moderate or severe clinical symptoms and avoid potentially life-threatening treatment-related complications. In the future, it is important to establish algorithms for implementing quantitative analysis of ^18^F-FDG PET/CT into daily clinical use.

## Figures and Tables

**Table 1:** Patient characteristics

Patients	Total cohort	UWCCC	OIL	*p* *
	*N* = 58 (%)	*N* = 29 (%)	*N* = 29 (%)	
Age; mean (±sd) (yr)	61 (14)	57 (15)	65 (13)	0.02
Gender				1
Male	32 (55%)	16 (55%)	16 (55%)	
Actionable mutation				0.4
BRAF wild type	38 (66%)	18 (62%)	20 (69%)	
BRAF mutated	10 (17%)	5 (17%)	5 (17%)	
Mutations other than BRAF	7 (12%)	6 (21%)	1 (4%)	
Unknown	3 (5%)	0 (0%)	3 (10%)	
ECOG performance status				0.001
0	30 (51%)	17 (59%)	13 (45%)	
1	15 (26%)	3 (10%)	12 (41%)	
2	4 (7%)	0 (0%)	4 (14%)	
Unknown	9 (16%)	9 (31%)	0 (0%)	
Anatomic site of primary				0.6
Cutaneous	48 (83%)	21 (73%)	27 (94%)	
Mucosal	4 (7%)	3 (10%)	1 (3%)	
Ocular	2 (3%)	2 (7%)	0 (0%)	
Unknown primary	4 (7%)	3 (10%)	1 (3%)	
Treatment setting				0.001
Adjuvant setting	10 (17%)	10 (34%)	0 (0%)	
Metastatic setting	48 (83%)	19 (66%)	29 (100%)	
Best response — only for metastatic disease (*N*=48)				0.8
Complete response	17 (35%)	8 (42%)	9 (31%)	
Progressive disease	10 (21%)	5 (26%)	5 (17%)	
Radiographic response	14 (29%)	3 (16%)	11 (38%)	
Mixed radiographic response	6 (13%)	2 (11%)	4 (14%)	
Other	1 (2%)	1 (5%)	0 (0%)	
Number of ^18^F-FDG PET/CT per patient, median (range)	4 (2–16)	3 (2–16)	4 (2–11)	0.5

*Sd* standard deviation, *UWCCC* University of Wisconsin Carbone Cancer Centre, *OIL* Institute of Oncology Ljubljana Slovenia,.

* *p* value was calculated using Fisher’s exact test to compare the statistical difference in patients characteristics between UWCCC and OIL
